# Portuguese Version of COVID-19 Perceived Risk Scale and COVID-19 Phobia Scale: Psychometric Properties

**DOI:** 10.3390/ejihpe11030078

**Published:** 2021-09-11

**Authors:** Ângela Leite, Diogo Guedes Vidal, Hélder Fernando Pedrosa e Sousa, Maria Alzira Pimenta Dinis, José Magano

**Affiliations:** 1 School of Human and Social Sciences (ECHS), University of Trás-os-Montes and Alto Douro (UTAD), Quinta de Prados, 5001-801 Vila Real, Portugal; angelal@utad.pt; 2 UFP Energy, Environment and Health Research Unit (FP-ENAS), University Fernando Pessoa (UFP), Praça 9 de Abril 349, 4249-004 Porto, Portugal; diogoguedesvidal@hotmail.com; 3 Department of Mathematics (DM. UTAD), University of Trás-os-Montes and Alto Douro, Quinta de Prados, 5001-801 Vila Real, Portugal; hfps@utad.pt; 4 Research Center in Business and Economics (CICEE), Universidade Autónoma de Lisboa, Rua Sta. Marta 47, 5° Andar, 1150-293 Lisboa, Portugal; 5 ISCET—Higher Institute of Business Sciences and Tourism, Rua de Cedofeita, 285, 4050-180 Porto, Portugal

**Keywords:** COVID-19 Perceived Risk Scale (C19PRS), COVID-19 Phobia Scale (C19PS), Coronavirus Anxiety Scale (CAS), Fear of COVID-19 Scale (FCV-19S)

## Abstract

The COVID-19 pandemic scenario has a psychological impact on individuals and society. A higher level of perceived risk concerning COVID-19 has been found when compared to other potential health threats. A misperception of risk in contrast with the real risk may lead people to develop disruptive cognitive, affective, or behavioral responses to the COVID-19 pandemic, namely, coronaphobia. Validated instruments are needed to evaluate such responses. This work aims to validate the COVID-19 Perceived Risk Scale (C19PRS) and the COVID-19 Phobia Scale (C19PS) in the Portuguese population. The two scales were translated from English to Portuguese using the back-translation technique. The cultural adaptation was framed in the context of establishing the validity and reliability of the instruments. In two studies, C19PRS and C19PS were validated for the adult Portuguese population (*N* = 1122; women = 725 (64.6%); mean age of 31.91 years old) through exploratory factorial analysis, followed by a confirmatory factorial analysis. Convergent validity was calculated by composite reliability (CR) and average variance extracted (AVE) values. Discriminant validity was assessed by square roots of the AVE values and their comparison with the C19PRS and C19PS dimensions’ cross-correlations. Both C19PRS and C19PS present a good adjustment model and solid reliability and validity and have significant correlations with fear of COVID-19 and COVID-19 anxiety scales.

## 1. Introduction

Coronaviruses are a large family of viruses known to cause diseases ranging from the common colds to more serious diseases, such as Middle Eastern Respiratory Syndrome (MERS) and Severe Acute Respiratory Syndrome (SARS) [1]. A new coronavirus outbreak started in Wuhan city, Hubei Province, China, in December 2019 and January 2020, and has spread widely across the world since then. The Coronavirus disease 2019 (COVID-19) evolved as a public health emergency due to its spread within most countries, and was declared a pandemic by the World Health Organization (WHO) on 11 March 2020 [2]. This Severe Acute Respiratory Syndrome Coronavirus-2 (SARS-CoV-2) has infected 219,189,790 individuals globally, with 4,544,789 deaths, as of 3 September 2021. In Portugal, the pandemic of COVID-19 has infected 1,042,322 infected individuals, with 17,766 mortalities [3].

Being highly infectious, the virus has put pressure on health systems everywhere, stretching them beyond their capacity and pushing them to disruption [4]. While authorities strive to monitor outbreak trends and mitigate the pandemic’s impact on health systems, the economy has been suffering a strong negative impact worldwide, especially in countries relying on services [5]. This is also the case in Portugal, where whole industries and jobs are threatened, such as the travel and tourism-related businesses, which accounted for more than 19% of exports and over 8% of gross domestic product (GDP) in 2019 [6]. This unsettling scenario has several direct and indirect effects, including on physical and mental health [7,8,9]. The outbreak has a psychological impact on individuals and society [10], as has been reported in different studies, namely, regarding depression, anxiety, stress [7,11], risk-attitudes [12], fear [13], perceived risk [14] and coronophobia [15]. In fact, Deng and colleagues [9] found that 45% of COVID-19 patients experience depression, 47% anxiety and 34% sleep disturbances. Additionally, Wang and colleagues [16] found that the most common psychological problems were somatization symptoms, depression, anxiety, problems of insomnia, and self-mutilating or suicidal thoughts. Cielo and colleagues [11] found a negative psychological impact and a high vulnerability of the young in the development of psychological distress, highlighting the need for structured and tailored psychological support. However, Prati and Mancini [17] stated that the psychological impact of COVID-19 lockdowns is small in magnitude and highly heterogeneous, suggesting that most people are psychologically resilient to their effects. In Portugal, there are almost no validated instruments specifically designed from scratch to assess the psychological impact of COVID-19. The aim of this study is to validate the COVID-19 Perceived Risk Scale (C19PRS) and the COVID-19 Phobia Scale (C19PS) in the Portuguese population.

Risk perception is the subjective assessment that people perform about something, concerning its characteristics, severity, and ways in which the risk can be managed [18]. A higher level of perceived risk was found in relation to COVID-19 as compared to other potential health threats [19]. According to Slovic [20], the characteristics of risk perception include (1) voluntariness (the risk of COVID-19 is imposed by external forces and is uncontrollable, thus being perceived as greater); (2) knowledge (COVID-19 is mostly unknown and unusual, making the perceived risk more frightening); (3) visibility (COVID-19 is an invisible risk, making it perceived as more hazardous than a visible one); (4) trust (the unknown nature of the disease, its repercussion in the mass media) [19]. The high numbers of infected and dead people, and the difficulty in having a common strategy, decrease people’s confidence in the management of the pandemic, which leads to a higher perception of risk. COVID-19 constitutes itself as a risk event that is related not only to people’s event-related risk perception, but also to their general risk perception [21]. Bruine de Bruin [22] stated that older age was associated with perceiving larger risks of dying if getting COVID-19, but with perceiving less risk of getting COVID-19, getting quarantined, or running out of money, as well as less depression and anxiety. Accordingly, He and colleagues [23] found that the perceived risk of getting COVID-19 increased by 4.9% for every one-year increase in age. Siegrist et al. [24] considered that perceived risks are important drivers for the acceptance of the government’s implemented measures to control COVID-19, and for more precautionary behavior.

A misperception of risk in relation to the real risk may lead people to the development of disruptive cognitive, affective, or behavioral responses to the COVID-19 pandemic, namely, coronaphobia. Fear is an adaptive animal defense mechanism fundamental for survival; however, if it is chronic or disproportionate, it may become harmful and can lead to the development of various psychiatric disorders [25]. According to Amin [26], coronaphobia is a persistent and excessive fear of the novel coronavirus, being classified as a particular type of DSM-5-specific phobia. Arpaci et al. [15] stated that natural disasters, such as the COVID-19 pandemic, can trigger phobic conditions with severe consequences for mental health. Lee et al. [27] found that coronaphobia explained additional variance in depression, generalized anxiety, and death anxiety, above sociodemographics, COVID-19 factors, and the vulnerability factors of neuroticism, health anxiety, and reassurance-seeking behaviors. Additionally, Lee and Crunk [28] reported that neuroticism, coronaphobia, and hypochondriasis were fear factors that predicted pandemic-related psychopathology in adults. Toprak et al. [29] found that individuals who stayed at home had higher coronaphobia than individuals who continued to work during the 3-month COVID-19 pandemic lockdown. According to Cihan and Durmaz [30], phobia scores were significantly higher in women, singles, people living alone, those living in an apartment, and those with higher economic and educational levels. Durmu and Durar [31] found a significant negative relationship between spiritual meaning and individuals’ fear of coronavirus, and between peace and fear of coronavirus. Individuals’ spirituality increases as fear of coronavirus decreases. Aware of the negative psychological impact of the pandemic on people, and aware of the importance of determining the specificity of that impact, Yıldırım and Güler [14] developed the COVID-19 Perceived Risk Scale (C19PRS), and Arpaci et al. [15] conceived the COVID-19 Phobia Scale (C19PS). Besides this, Yildirim et al. [32] found that perceived risk and fear can significantly increase engagement in preventive behaviors. However, Yildirim and Guler [33] found that COVID-19 perceived risk increases death distress and reduces happiness. Arpaci and colleagues [34] found a significant positive correlation between coronaphobia and state anxiety. Additionally, Arpaci and colleagues [35] found that people were afraid of being infected, and those who got infected were afraid of death. In Portugal, there are no validated instruments to assess these dimensions; therefore, the aim of this study is to adapt the C19PRS and the C19PS to the Portuguese population.

## 2. Materials and Methods

### 2.1. Sample

Two independent samples were used. The total sample consisted of 1122 participants, with a mean age of 31.91, who were mostly women without university educations and were professionally active (Table 1). The EFA sample (*n* = 561) did not differ from the CFA sample (*n* = 561) concerning sociodemographic issues (assessed by chi-square tests and Student’s *t*-tests), except for age (*t*(1111, 613) = 2.22; *p* = 0.027; *d* = 0.13), as the EFA sample was older (*M* = 32.82 years old; *SD* = 14.32) than the CFA sample (*M* = 31.00 years old; *SD* = 13.12).

### 2.2. Procedures

The original authors of the C19PRS [14] and the C19PS [15] gave permission to validate the instrument in a Portuguese population. The two scales were then translated from English to Portuguese using the back-translation technique [36]. The process of back-translation included the translation of the previous translation back into its original language, and then the comparison of the new translation with the previous one, checking whether the translation conveys the same message as the source text, noting the issues that may compromise the translation quality, and informing about the parts of the translated document that may need to be revised again. Cultural adaptation is framed in the context of establishing the validity and reliability of the instruments [37]. The translations of both instruments are available as Appendix A and Appendix B (Table A1 and Table A2). The protocol (including the sociodemographic questionnaire; C19PRS, C19PS, FCV-19S and CAS) was submitted to the ethics committee of the University of Trás-os-Montes e Alto Douro, having been approved on 1 September 2020 (no specific reference assigned, date acting as reference ID) and made available online. It was released to the general population through a page dedicated to the study on a social network, with data being collected between 1 October and 30 November 2020. All procedures performed in this study adhered to the institutional research committee’s ethical standards, and the 1964 Helsinki declaration [38] and its later amendments. Before the investigation protocol, participants were informed about the research content and its objectives. Confidentiality and anonymity were guaranteed, and informed consent was obtained.

### 2.3. Instruments

#### 2.3.1. Sociodemographic Questionnaire

The questionnaire included questions about gender (man vs. woman), age (numerical), education (no university studies vs. university studies), and professional status (inactive—unemployed, sick, retired, on medical leave—vs. active—students, employees, housewives).

#### 2.3.2. COVID-19 Perceived Risk Scale (C19PRS)

The authors [14] conceived the COVID-19 related perceived risk by adapting an 8-item Severe Acute Respiratory Syndrome (SARS) Risk Perception Scale [39], answered on a 5 point Likert scale (1—negligible to 5—very large). The authors [14] changed the wording of the original items, for example, replacing “SARS” with “COVID-19” and “cancer/accidents” with “diabetes/asthma”. Examples of items are: “What is the likelihood that you would die from the COVID-19?” and “How worried are you about contracting the COVID-19?”. The scale includes a cognitive dimension and an emotional one of personal risk. Higher scores indicate higher levels of perceived risk related to COVID-19. The authors also found that the reliability ranged from 0.70 to 0.74 for the cognitive dimension and from 0.84 to 0.88 for the emotional dimension, suggesting satisfactory internal consistency reliability for the C19PRS.

#### 2.3.3. COVID-19 Phobia Scale (C19PS)

The authors [15] developed this scale to detect COVID-19 phobia early to intervene as soon as possible. They considered the possibility of this phobia developing due to the psychological burden caused by COVID-19. The authors [15] developed a structure of 20 items answered on a 5-point Likert scale (1—strongly disagree to 5—strongly agree) and 4 subscales (psychological, psycho-somatic, economic, and social factors). Examples of items are: “The fear of coming down with coronavirus makes me very anxious” and “I am extremely afraid that someone in my family might become infected by the coronavirus”. They also found that the reliability ranged from 0.85 to 0.90 for the four factors and 0.92 for total, suggesting the good internal consistency and reliability of the C19PS.

#### 2.3.4. Fear of COVID-19 Scale (FCV-19S)

According to Ahorsu et al. [40], the Fear of COVID-19 Scale (FCV-19S) was developed to complement the clinical efforts made in preventing the spread of and treating COVID-19 cases. The scale was validated in the Portuguese population by Magano et al. [13]. This is a seven-item scale, answered on a 5-point Likert scale (1—strongly disagree to 5—strongly agree), with robust psychometric properties (composite reliability—0.88; average variance extracted—0.5; internal consistency (Cronbach’s α)—0.82), making it reliable and valid in assessing fear of COVID-19 among the general population and useful in allaying COVID-19 fears among individuals [40]. Examples of items are: “I am most afraid of coronavirus-19”, and “It makes me uncomfortable to think about coronavirus-19”.

#### 2.3.5. Coronavirus Anxiety Scale (CAS)

This scale was developed with the purpose of studying the mental health response to COVID-19 [41]. The scale was validated in the Portuguese population by Magano et al. [13]. According to the author [41], an instrument to quickly identify cases of dysfunctional anxiety and symptom severity associated with the coronavirus was needed. This is a five-item scale, answered in a 5-point Likert scale (0—not at all to 4—nearly every day over the last 2 weeks) that assesses distinct physiological reactions of anxiety related to the coronavirus, which was highly reliable as a cluster (*α* = 0.93) [41]. Examples of the items are: “I felt dizzy, lightheaded, or faint, when I read or listened to news about the coronavirus”, and “I had trouble falling or staying asleep because I was thinking about the coronavirus”.

### 2.4. Analytic Approach

One-half of the study’s data were used for exploratory factor analysis (EFA) and the other half for confirmatory factor analysis (CFA), to address the sampling error influences. The EFA was used to test the authors’ models, while a CFA was used to test the EFA results’ replicability. Convergent validity was calculated by composite reliability (CR) and average variance extracted (AVE) values. Discriminant validity was assessed by square roots of the AVE values and their comparison with the C19PRS and C19PS dimensions’ cross-correlations. EFA was assessed by correlation matrix (>*r* = 0.30 and <*r* = 0.90) (Yong and Pearce [42]), determinant score > 0.00001, Bartlett’s Test of Sphericity (*p* < 0.001), the Kaiser–Meyer–Olkin measure (KMO) of sampling adequacy (>0.50), diagonal element of the anti-correlation matrix (>0.50), maximum likelihood factor analysis (>0.40), Kaiser’s criterion of eigenvalues (>1) [42], structure coefficients (>0.40), communality coefficients (<0.40) and Cronbach’s alpha (>0.70). CFA was assessed by Chi-square (*χ*^2^ < 2.00), Comparative Fit Index (CFI > 0.90), Tucker–Lewis Index (TLI > 0.90), root mean square error of approximation (RMSEA < 0.06), PCLOSE (>0.05) and standardized root mean square residual (SRMR < 0.06) [43]. The AVE should be equal to or greater than 0.50, the CR greater than 0.70, and the square roots of AVE should be greater than the correlations between constructs [44]. Statistical analysis was performed using SPSS version 27.0, and confirmatory factor analysis (CFA) was run using AMOS version 27.0.

## 3. Results

### 3.1. Study 1

#### 3.1.1. Exploratory Factorial Analysis (EFA) Results: COVID-19 Perceived Risk Scale (C19PRS)

Several procedures have been carried out to determine whether the data are suitable for EFA: in the correlation matrix, items three and four presented several correlations below *r* = 0.30 with other items; so, according to Yong and Pearce [45], they should be removed, as they indicated a lack of patterned relationships. None of the correlations between items were above *r* = 0.90, suggesting a lack of multicollinearity; also, the determinant score was above the rule of thumb of 0.00001 (0.04), indicating an absence of multicollinearity. Bartlett’s Test of Sphericity (1753.124; default freedom = 28; *p* < 0.001) confirmed that our items had patterned relationships amongst the variables (*p* < 0.001). Finally, looking at the Kaiser–Meyer–Olkin measure (KMO) of sampling adequacy (cut-off above 0.50; in our study 0.77) and the diagonal element of the anti-correlation matrix (cut-off of above 0.50), which ranged between 0.64 and 0.84, it was found that the sample was suitable for EFA. The eight items of the C19PRS scale were subjected to an EFA with varimax rotation. In the maximum likelihood factor analysis, the cut-off point was 0.40 and the Kaiser’s criterion of eigenvalues was greater than one (Field, 2009), suggesting a two-factor solution and accounting for 62.15% of the total variance explained. The eight items were psychometrically robust (Table 2). The structure coefficients ranged from 0.67 to 0.84, and communality coefficients ranged from 0.47 to 0.74. These items were reliable as a single dimension (*α* = 0.80) (if item 3 was deleted, the alpha’s value increased to 0.82) and as two-factor dimensions (first one *α* = 0.85; second one *α* = 0.68).

#### 3.1.2. Confirmatory Factorial Analysis (CFA) Results: COVID-19 Perceived Risk Scale (C19PRS)

**Figure 1:**
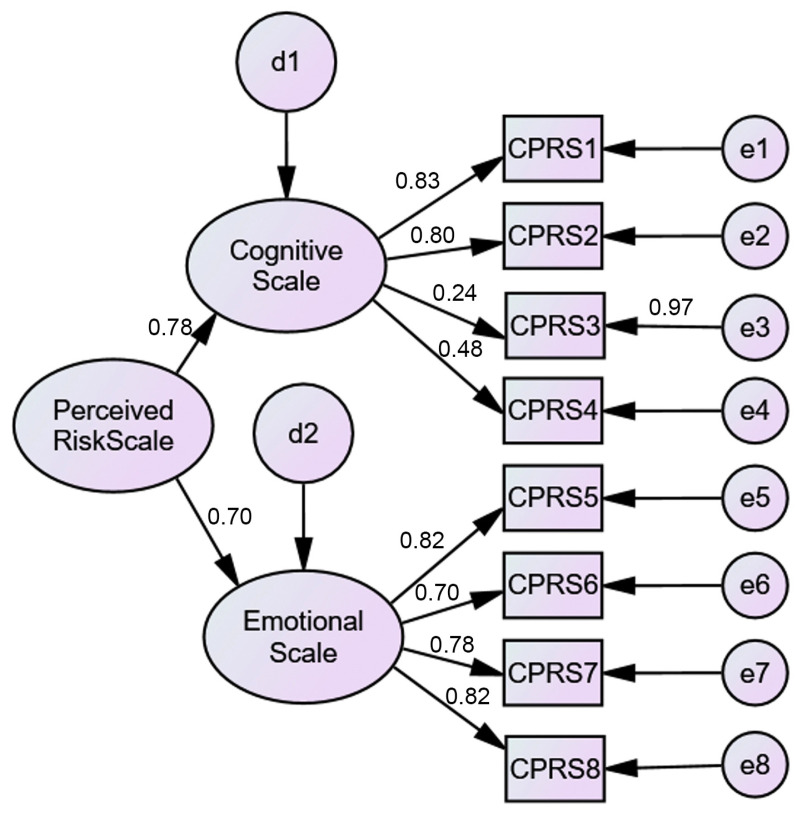
Two-factor (8 items) CFA C19PRS model.

**Figure 2:**
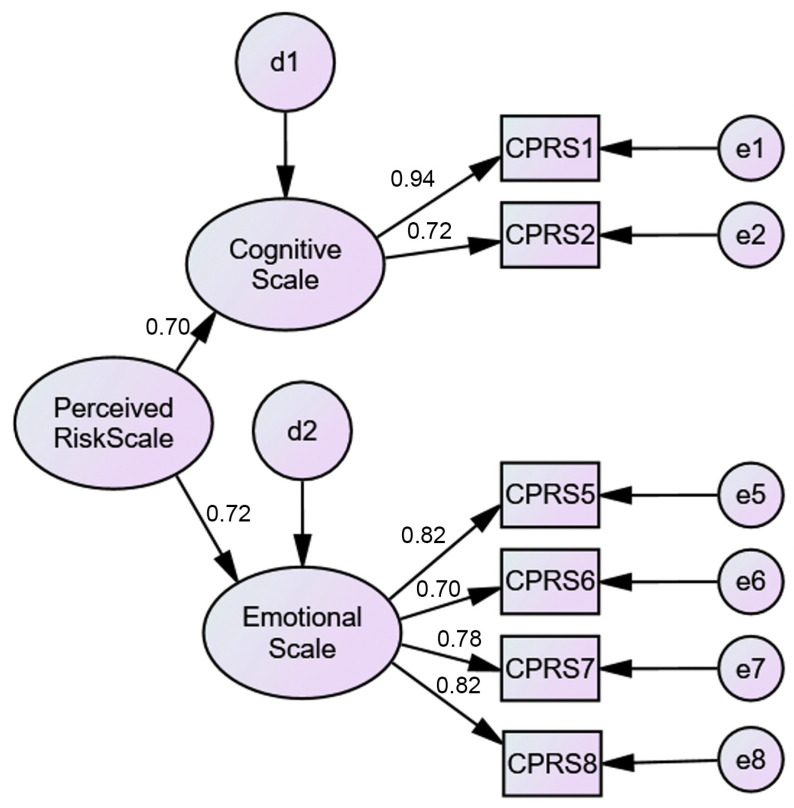
Two-factor (6 items) CFA C19PRS model.

A CFA was performed to test the model found in the EFA. The results did not support the EFA findings (Figure 1). A poor model fit was found (*χ*^2^(19) = 114.59, *p* < 0.001) with a poor fit for all of indices (*χ*^2^/*df* ratio = 6.03; CFI = 0.94; TLI = 0.92; SRMR = 0.06; RMSEA = 0.10; PCLOSE = 0.00). When modification indices were analyzed, it was found that the establishment of several correlations was suggested, the first one being the correlation between the error of item 3 and the error of item 4. However, the standardized regression weight of item 3 was 0.24, which is well below the recommended value (≥0.50). The same happened with item 4. The decision to remove items 3 and 4 from the model was made (Figure 2). This model presented a good fit (*χ*^2^(8) = 12.57, *p* < 0.001) with a god fit for all of indices (*χ*^2^/*df* ratio = 1.57; CFI = 0.99; TLI = 0.99; SRMR = 0.01; RMSEA = 0.03; PCLOSE = 0.80) (Figure 2). Additionally, the value of Cronbach’s alpha for the total scale with six items was 0.84; for the COVID-19 emotional perceived risk scale (four items) it was 0.85, and for the COVID-19 cognitive perceived risk scale (two items) it was 0.81. To test whether the C19PRS construct was measured the same way across genders (women vs. men), a multigroup CFA was performed. The results demonstrated gender differences, which were evidenced by the model fit (*χ*^2^(16) = 35.09, *p* = 0.04) (*χ*^2^/*df* ratio = 2.19; CFI = 0.99; TLI = 0.98; SRMR = 0.02; RMSEA = 0.04; PCLOSE = 0.77). Women (*M* = 2.76; *SD* = 0.70) presented significantly higher levels of COVID-19 perceived risk than men (*M* = 2.39; *SD* = 0.76).

Construct validity was also confirmed through the convergence of validity with the Fear of COVID-19 Scale (FC19S) [13,40] and the COVID Anxiety Scale (CAS) [13,41]. FC19S and CAS were previously assessed in the Portuguese population, with confirmation of good psychometric quality, and so no translation process was necessary. The Spearman’s correlation coefficient was used to estimate the convergent validity (Table 3), as usual in similar cases. The C19PRS Total, Cognitive and Emotional correlated significantly with all the other dimensions, except C19PRS Cognitive, which did not correlate with CAS Total.

#### 3.1.3. Convergent and Discriminant Validity

Table 4 presents inter-item correlations along with convergent and discriminant validity coefficients. Convergent validity was calculated by assessing composite reliability (CR) and average variance extracted (AVE) values. The AVE and CR results are higher than the thresholds of 0.50 and 0.70, respectively [46], and each factor significantly correlated with the other (*p* < 0.01). The square roots of the AVE values (reported in the off-diagonal, Table 4) being higher than the cross-correlations, the discriminant validity of the Cl9PS was guaranteed.

### 3.2. Study 2

#### 3.2.1. Exploratory Factorial Analysis (CFA) Results: COVID-19 Phobia Scale (C19PS)

To determine whether the data are suitable for EFA, several procedures have been carried out: in the correlation matrix, items three and four presented several correlations below *r* = 0.30 with other items; so, according to Yong and Pearce [45], they had to be removed, as they indicated a lack of patterned relationships. No correlations between items were above *r* = 0.90, suggesting the lack of multicollinearity; also, the determinant score was above the rule of thumb of 0.00001 (0.04), indicating an absence of multicollinearity. Bartlett’s Test of Sphericity (6459.25; default freedom = 190; *p* < 0.001) confirmed that our items had patterned relationships amongst the variables (*p* < 0.001). Finally, looking at the Kaiser–Meyer–Olkin measure (KMO) of sampling adequacy (cut-off above 0.50; in our study 0.93) and the diagonal element of the anti-correlation matrix (cut-off of above 0.50), which ranged between 0.86 and 0.96, it was found that the sample was suitable for EFA. An EFA with varimax rotation was performed with the 20 items of the C19PS scale. The maximum likelihood factor analysis (with a cut-off point of 0.40 and the Kaiser’s criterion of eigenvalues greater than 1 [42]) suggested a four-factor solution, explaining 65.18% of total variance. The 20 items were psychometrically robust (Table 5). Structure coefficients were saturated from 0.60 to 0.87, and communality coefficients were saturated from 0.32 (item 6) to 0.85 (item 8). These items were reliable as a unique dimension (α = 0.92) and as a four-factor dimension (if item 6 was deleted from Factor 2, the alpha’s value increased to 0.87) (Table 5). In this analysis, the items’ distributions followed the proposal of the original version, except for item 20, which, in the original version, saturated in factor 4, and in this study, saturated in factor 1.

#### 3.2.2. Confirmatory Factorial Analysis (CFA) Results: COVID-19 Phobia Scale (C19PS)

**Figure 3:**
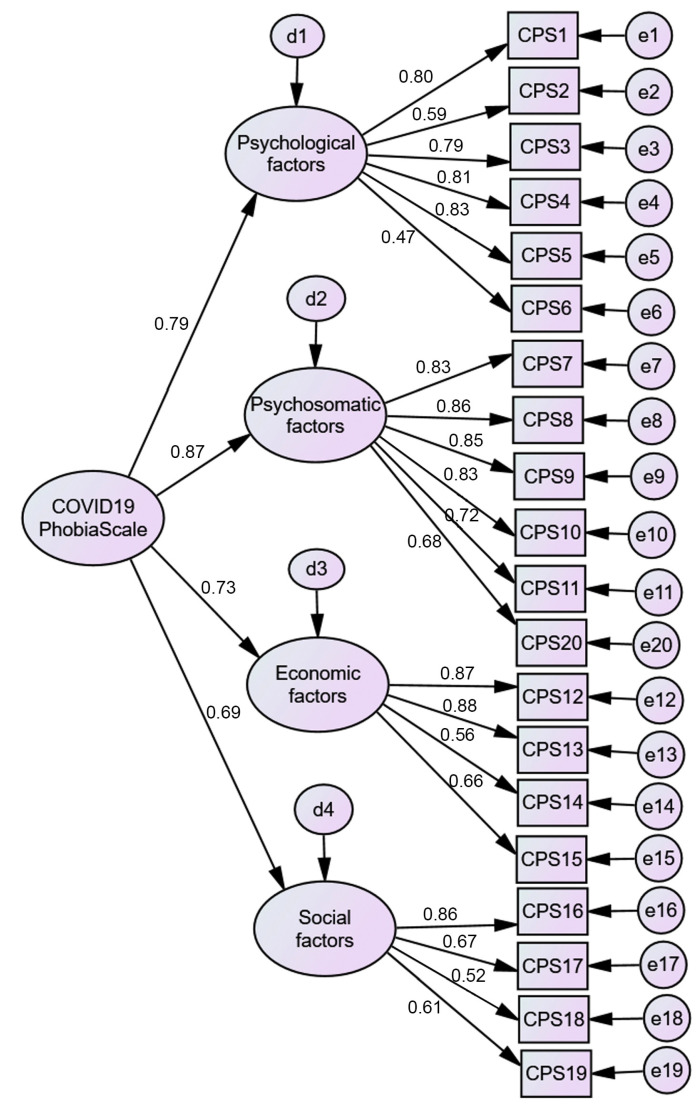
Two-factor (20 items) CFA C19PS model.

**Figure 4:**
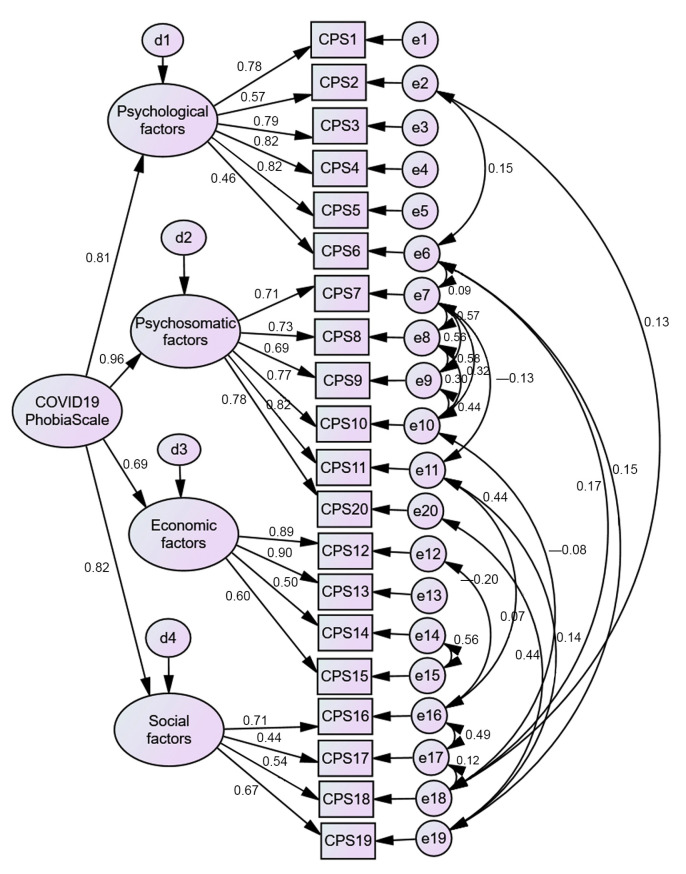
Two-factor (20 items) CFA C19PS model.

A CFA was performed to test the model found in EFA. The results did not support the EFA findings (Figure 3). A poor model fit was found (*χ*^2^(169) = 1130.27, *p* < 0.001) with a poor fit for all indices (*χ*^2^/*df* ratio = 6.69; CFI = 0.86; TLI = 0.84; SRMR = 0.09; RMSEA = 0.10; PCLOSE = 0.00). When the modification indices were analyzed, several correlations between item errors were suggested; most of them were established within each factor, but some occurred between item errors within different factors. From a theoretical point of view, in all cases, there was content proximity. After the correlations were established, this model presented a good fit (*χ*^2^(149) = 379.67, *p* < 0.001) with a god fit for almost all indices (*χ*^2^/*df* ratio = 2.55; CFI = 0.97; TLI = 0.96; SRMR = 0.01; RMSEA = 0.05; PCLOSE = 0.25) (Figure 4). To test if the C19PS construct was measured the same way across genders (women vs. men), a multigroup CFA was performed. The results show gender differences (*χ*^2^(16) = 547.91, *p* = 0.01) (*χ*^2^/*df* ratio = 1.84; CFI = 0.96; TLI = 0.95; SRMR = 0.02; RMSEA = 0.04; PCLOSE = 1.00). Women (*M* = 2.72; *SD* = 0.64) presented significantly higher levels of COVID-19 phobia than men (*M* = 2.48; *SD* = 0.64).

Construct validity was also confirmed through the convergence of validity with the C19PRS, the Fear of COVID-19 Scale (FCV-19S) [13,40], the C19PRS and the COVID Anxiety Scale (CAS) [13,41]. Spearman’s correlation coefficient was used to estimate the convergent validity (Table 6). C19PS Total, Psycho-somatic, Economic and Social factors correlated significantly with all the other dimensions; C19PS Psychological factors correlated significantly with almost all other dimensions, except the C19PRS Cognitive factor.

#### 3.2.3. Convergent and Discriminant Validity

Table 7 presents inter-item correlations along with convergent and discriminant validity coefficients. Convergent validity was assessed via the composite reliability (CR) and average variance extracted (AVE) values. The AVE and CR are higher than the thresholds of 0.50 and 0.70, respectively [46], except for the AVE in the psychosomatic and social factors. Each factor significantly correlated with the other factors (*p* < 0.01). All square roots of the AVE values (in the off-diagonal, Table 7) were higher than the cross-correlations; so, discriminant validity of the Cl9PS was guaranteed.

## 4. Discussion

The psychological impact of the COVID-19 pandemic has been studied by different authors with the aim of improving the psychological assessment and intervention of people most affected by COVID-19. To this end, validated instruments are needed to diagnose and intervene in a timely and appropriate manner. Therefore, the aim of this study was to validate two instruments for the general Portuguese population, using a sample of Portuguese adults: COVID-19 Perceived Risk Scale (C19PRS) [14] and COVID-19 Phobia scale (C19PS) [15].

The validation of the C19PRS did not confirm the original structure proposed by the authors (eight items evenly distributed by two factors). In fact, the structure of this construct, in the Portuguese version, excludes two items (item 3 and item 4) from factor 1 (cognitive), with factor 2 (emotional) remaining the same as the original. In the Portuguese version, it is suggested that the C19PRS be a multidimensional scale, including cognitive and emotive aspects of perceived personal risk related to COVID-19. The Portuguese version showed good internal consistency, reliability, convergent validity, and evidence of gender differences in the C19PRS total and subscales.

C19PRS correlates positively and significantly with fear, anxiety, and phobia related to COVID-19: the higher the perceived risk of contracting COVID-19, the higher the fear, anxiety, and phobia in relation to COVID-19. These results corroborate those of Amin [26], Arpaci et al. [15], Cori et al. [18], Ho et al. [10], Lee [41], Lee and Crunk [28], Lee et al. [27], Magano et al. [13], Paulino et al. [7], Wang et al. [47], Yıldırım and Güler [14] and Zhong et al. [19]. Deng and colleagues [9] found that most COVID-19 patients experience depression, anxiety, and sleep disturbances. Cielo and colleagues [11] found a negative psychological impact on, and high vulnerability in, the young in the context of developing psychological distress. The adaptation of the C19PS [15] confirmed the original structure proposed by the authors (20 items distributed by four factors—psychological, psychosomatic, economic, and social factors), although item 20 (“I am unable to curb my anxiety of catching coronavirus from others”), in the original version, saturated in factor 4 (social factor), and in this study, it saturated in factor 1 (psychosomatic factor). In fact, the content of the item can be referred to as a social factor (*getting the virus from others*), but it can also be addressed as a psychosocial factor (*I can’t control the anxiety*). In our study, this item clearly saturated in the psychosomatic factor and, in addition, if it was removed from this factor, the value of the Cronbach’s alpha decreased. Therefore, the authors decided to keep it in this factor. Good model adjustment was obtained only after the establishment of several correlations between item errors. The Portuguese version showed good internal consistency, reliability, convergent validity, and evidence of gender differences in the C19PS total and its subscales. C19PS correlates positively and significantly with fear, anxiety, and risk perception related to COVID-19: the higher the phobia of COVID-19, the higher the fear, anxiety, and risk perception in relation to COVID-19. These results are also in line with those of Amin [26], Arpaci et al. [15], Ho et al. [10], Lee [41], Lee and Crunk [28], Lee et al. [27], Magano et al. [13], Paulino et al. [7], Toprak Celenay et al. [29], and Wang et al. [47].

Gender differences were found in the values of C19PRS and C19PS, with women scoring higher, which is in line with the results of Cihan and Durmaz [30], who found phobia scores significantly higher in women. Additionally, Rodriguez-Besteiro et al. [48] found that females presented a higher perception of danger (assessed by the risk perception scale) related to the COVID-19 virus than males. Concerning age, Bruine de Bruin [22] and He and colleagues [23] found that older people perceived larger risks of dying from getting COVID-19, but perceived less risk of getting COVID-19, getting quarantined, or running out of money, as well as less depression and anxiety.

The implications of this study include the empowerment of Portuguese psychologists, who now have an instrument to assess COVID-19-related risk perception and phobia. Future studies with these instruments should be based on representative samples of the Portuguese population.

This study presents some limitations: (1) the participants belong to the general Portuguese population, but they are not representative of it; (2) the perceived risk and phobia related to COVID-19 are self-reported, and some respondents may have provided the desired social responses; (3) the professional distribution in the present study was unequal, with 83.1% active, which might affect the psychometric evaluation; finally, (4) convenience sampling may skew the results.

## 5. Conclusions

This study demonstrated that the C19RPS and the C19PS are robust instruments with good psychometric properties to assess, respectively, risk perception and phobia related to COVID-19 in the Portuguese population. Henceforth, Portuguese psychologists have an instrument to assess COVID-19-related risk perception and phobia, validated for the general Portuguese population.

## Figures and Tables

**Table 1:** Total sample characteristics (*n* = 1122).

Variables	*M*	*SD*
Age	31.91	13.76
	*N*	%
Gender		
Male	397	35.4%
Female	725	64.6%
Education		
University studies	495	44.1%
Other	627	55.9%
Professional status		
Active	932	83.1%
Inactive	190	16.9%

Notes: *M* = Mean; *SD* = Standard deviation; *N* = Frequencies; % = Percentage.

**Table 2:** C19PRS’s EFA results.

		LD1	LD2	*h* ^2^	*M*	*SD*
Item 1	Likelihood of acquiring COVID-19		0.67	0.58	2.30	0.92
Item 2	Likelihood of acquiring COVID-19 compared to other persons		0.76	0.64	2.05	0.90
Item 3	Likelihood of acquiring other diseases		0.67	0.47	1.13	1.05
Item 4	Likelihood of dying from COVID-19		0.69	0.50	0.97	0.99
Item 5	Worried about acquiring COVID-19	0.79		0.70	2.35	1.06
Item 6	Worried about a family member contracting COVID-19	0.82		0.67	3.32	0.85
Item 7	Worried about COVID-19 occurring in your region	0.81		0.68	2.58	1.05
Item 8	Worried about COVID-19 emerging as a health issue	0.84		0.74	2.79	1.02
Total	CPRS				2.19	0.63
Factor 1	5, 6, 7, 8 items	Emotional scale			2.76	0.83
Factor 2	1, 2, 3, 4 items	Cognitive scale			1.61	0.69

Notes: LD = Structure coefficients; *h*^2^ = Extracted communality coefficients; *M* = Mean; *SD* = Standard deviation.

**Table 3:** Spearman correlations between C19PRS dimensions and FC19S and CAS.

	1	2	3	4	5	6	7
1 C19PR Total	1						
2 C19PR Cognitive	0.66 **	1					
3 C19PR Emotional	0.85 **	0.35 **	1				
4 FCV-19S Total	0.55 **	0.18 **	0.58 **	1			
5 FC19S Emotional fear	0.60 **	0.20 **	0.64 **	0.92 **	1		
6 FC19S Cognitive fear	0.34 **	0.12 **	0.34 **	0.83 **	0.58 **	1	
7 CAS Total	0.24 **	00.07	0.29 **	0.50 **	0.37 **	0.51 **	1

Notes: C19PRS—COVID-19 Perception Risk Scale; FC19S—Fear of COVID-19 Scale; CAS—COVID-19 Anxiety Scale. ** The correlation is significant at the 0.01 level.

**Table 4:** C19PRS convergent and discriminant validity.

		AVE	CR	F1	F2
Factor 1	Cognitive factor	0.80	0.89	0.89 **	
Factor 2	Emotional factor	0.67	0.89	0.43 **	0.82 **

Notes: ** *p* < 001; AVE = average variance extracted; CR = composite reliability.

**Table 5:** C19PS’s EFA results.

		LD1	LD2	LD3	LD4	*h* ^2^	*M*	*SD*
Item 1	Psychological factors 1		0.70			0.67	3.33	1.05
Item 2	Psychological factors 2		0.74			0.58	4.23	0.88
Item 3	Psychological factors 3		0.72			0.64	3.24	1.04
Item 4	Psychological factors 4		0.77			0.68	3.59	1.00
Item 5	Psychological factors 5		0.74			0.68	3.32	1.07
Item 6	Psychological factors 6		0.44			0.32	3.26	1.11
Item 7	Psycho-somatic factors 1	0.84				0.77	1.75	0.87
Item 8	Psycho-somatic factors 2	0.87				0.85	1.81	0.95
Item 9	Psycho-somatic factors 3	0.87				0.83	1.69	0.85
Item 10	Psycho-somatic factors 4	0.80				0.73	1.85	0.98
Item 11	Psycho-somatic factors 5	0.60				0.57	2.20	1.13
Item 12	Economic factors 1			0.80		0.79	2.33	1.05
Item 13	Economic factors 2			0.73		0.74	2.41	1.08
Item 14	Economic factors 3			0.68		0.60	1.65	0.83
Item 15	Economic factors 4			0.70		0.69	1.77	0.88
Item 16	Social factors 1				0.65	0.62	2.80	1.06
Item 17	Social factors 2				0.80	0.67	2.84	1.12
Item 18	Social factors 3				0.55	0.43	3.19	1.13
Item 19	Social factors 4				0.62	0.55	3.18	1.19
Item 20	Social factors 5 (original study) Psycho-somatic factors 5 (this study)	0.53				0.62	2.35	1.07
						*α*	*M*	*SD*
Total	C19PS					0.92	2.64	0.65
Factor 1	7, 8, 9, 10, 11, 20 items	Psychological factors				0.91	1.94	0.81
Factor 2	1, 2, 3, 4, 5, 6 items	Psychosomatic factors				0.86	3.50	0.78
Factor 3	12, 13, 14, 15 items	Economic factors				0.82	2.04	0.78
Factor 4	16, 17, 18, 19 items	Social factors				0.73	3.00	0.84

Notes: LD = Structure coefficients; *h*^2^ = Extracted communality coefficients; *M* = Mean; *SD* = Standard deviation; α = Cronbach’s alpha.

**Table 6:** Spearman correlations between C19PS dimensions and FCV-19S, C19PRS and CAS.

	1	2	3	4	5	6	7	8	9	10	11	12
1 C19PS Total	1											
2 C19PS Psychological	0.84 **	1										
3 C19PS Psychosomatic	0.84 **	0.59 **	1									
4 C19PS Ecomonic	0.73 **	0.60 **	0.46 **	1								
5 C19PS Social	0.72 **	0.45 **	0.57 **	0.39 **	1							
6 FC19S Total	0.75 **	0.70 **	0.64 **	0.52 **	0.51 **	1						
7 FC19S Emotional fear	0.69 **	0.57 **	0.67 **	0.44 **	0.50 **	0.92 **	1					
8 FC19S Cognitive fear	0.65 **	0.73 **	0.44 **	0.52 **	0.37 **	0.83 **	0.58 **	1				
9 C19PRS Total	0.52 **	0.36 **	0.56 **	0.28 **	0.43 **	0.55 **	0.60 **	0.34 **	1			
10 C19PRS Cognitive	0.14 **	00.08	0.16 **	0.09 *	0.13 **	0.18 **	0.20 **	0.12 **	0.66 **	1		
11 C19PRS Emotional	0.60 **	0.40 **	0.67 **	0.28 **	0.51 **	0.58 **	0.64 **	0.34 **	0.85 **	0.35 **	1	
12 CAS Total	0.44 **	0.46 **	0.36 **	0.29 **	0.29 **	0.50 **	0.37 **	0.51 **	0.24 **	0.07	0.29 **	1

Notes: C19PS—COVID-19 Phobia Scale; FC19S—Fear of COVID-19 Scale; C19PRS—COVID-19 Perception Risk Scale; CAS—COVID-19 Anxiety Scale. ** The correlation is significant at the 0.01 level. * The correlation is significant at the 0.05 level.

**Table 7:** C19PS convergent and discriminant validity.

		AVE	CR	F1	F2	F3	F4
Factor 1	Psychological factors	0.58	0.89	0.76 **			
Factor 2	Psychosomatic factors	0.48	0.84	0.582 **	0.69 **		
Factor 3	Economic factors	0.53	0.82	0.605 **	0.479 **	0.73 **	
Factor 4	Social factors	0.43	075	0.490 **	0.612 **	0.432 **	0.66 **

Notes: ** *p* < 0.001; AVE = average variance extracted; CR = composite reliability.

## Data Availability

The data presented in this study are available on request from the corresponding author.

## Appendix A. Coronavirus-19 Perceived Risk Scale

### Portuguese Version

Por favor, leia cada uma das afirmações a seguir e escolha a resposta que melhor o(a) descreve.

**Table A1:** Items of the Coronavirus-19 Perceived Risk Scale (Portuguese version).

	Negligenciável/Muito Pouco				Muito/Muito Grande
1. Qual considera que é a probabilidade de contrair COVID-19?	□	□	□	□	□
2. Qual considera que é a probabilidade de contrair COVID-19 em comparação com outras pessoas?	□	□	□	□	□
3. Qual considera que é a probabilidade de contrair outras doenças (por exemplo, diabetes/asma)?	□	□	□	□	□
4. Qual considera que é a probabilidade de morrer devido ao COVID-19?	□	□	□	□	□
5. Quão preocupado está com a possibilidade de contrair COVID-19?	□	□	□	□	□
6. Quão preocupado está com o facto de um familiar contrair COVID-19?	□	□	□	□	□
7. Quão preocupado está com a ocorrência de COVID-19 na sua região?	□	□	□	□	□
8. Quão preocupado está com facto do COVID-19 emergir como um problema de saúde?	□	□	□	□	□

## Appendix B. Coronavirus-19 Phobia Scale

### Portuguese Version

Por favor, assinale a sua concordância ou discordância em relação às seguintes afirmações:

**Table A2:** Items of the Coronavirus-19 Phobia Scale (Portuguese version).

	Discordo Completamente	Discordo	Não Concordo Nem Discordo	Concordo	Concordo Completamente
1. O medo de contrair o coronavírus deixa-me muito ansioso(a).	□	□	□	□	□
2. Tenho muito medo que alguém da minha família seja infetado pelo coronavírus.	□	□	□	□	□
3. Notícias sobre mortes relacionadas com o coronavírus causam-me grande ansiedade.	□	□	□	□	□
4. As incertezas à volta do coronavírus causam-me uma enorme ansiedade.	□	□	□	□	□
5. A velocidade com que o coronavírus se espalhou provoca-me um grande pânico.	□	□	□	□	□
6. Discuto apaixonadamente (ou quero discutir) com pessoas que considero que se comportam de maneira irresponsável face ao coronavírus.	□	□	□	□	□
7. Tenho sérias dores de estômago por sentir medo do coronavírus.	□	□	□	□	□
8. Sinto uma forte dor no peito devido ao medo do coronavírus.	□	□	□	□	□
9. Tenho tremores devido ao medo do coronavírus.	□	□	□	□	□
10. Tenho problemas de sono por causa do medo do coronavírus.	□	□	□	□	□
11. O coronavírus deixa-me tão tenso que me sinto incapaz de fazer o que fazia antes sem problemas.	□	□	□	□	□
12. A possibilidade de escassez de alimentos devido à pandemia do coronavírus causa-me ansiedade.	□	□	□	□	□
13. A possibilidade de falta de material de limpeza e higiene devido à pandemia de coronavírus causa-me ansiedade.	□	□	□	□	□
14. Eu estou a armazenar comida com medo do coronavírus.	□	□	□	□	□
15. Depois da pandemia de coronavírus, não me sinto relaxado(a) a menos que verifique constantemente os meus mantimentos em casa.	□	□	□	□	□
16. Após a pandemia de coronavírus, fico extremamente ansioso(a) quando vejo pessoas a tossir.	□	□	□	□	□
17. Após a pandemia de coronavírus, evito ativamente as pessoas que vejo a espirrar.	□	□	□	□	□
18. Após a pandemia do coronavírus, percebi que passo longos períodos de tempo a lavar as mãos.	□	□	□	□	□
19. O medo de contrair o coronavírus interfere seriamente nas minhas relações sociais.	□	□	□	□	□
20. Não consigo controlar a minha ansiedade de contrair o coronavírus através de outras pessoas.	□	□	□	□	□
